# Offen gemacht: Der Stand der internationalen evidenzbasierten Forschung zu Open Educational Resources (OER)

**DOI:** 10.1007/s11618-021-01043-2

**Published:** 2021-08-31

**Authors:** Daniel Otto, Nadine Schröder, Daniel Diekmann, Pia Sander

**Affiliations:** 1 Fakultät für Bildungswissenschaften, Lehrstuhl für Mediendidaktik und Wissensmanagement | Learning Lab, Universität Duisburg-Essen, Gebäude WST C08.15 (Weststadttürme), Berliner Platz 6–8, 45127 Essen, Deutschland; 2 Fakultät für Bildungswissenschaften, Lehrstuhl für Mediendidaktik und Wissensmanagement | Learning Lab, Universität Duisburg-Essen, Gebäude WST C.08.15 (Weststadttürme), Berliner Platz 6–8, 45127 Essen, Deutschland; 3 Fakultät für Bildungswissenschaften, Lehrstuhl für Mediendidaktik und Wissensmanagement | Learning Lab, Universität Duisburg-Essen, Gebäude WST C.08.12 (Weststadttürme), Berliner Platz 6–8, 45127 Essen, Deutschland; 4 Fakultät für Bildungswissenschaften, Lehrstuhl für Mediendidaktik und Wissensmanagement | Learning Lab, Universität Duisburg-Essen, Gebäude S06 S02 A43, Universitätsstraße 2, 45141 Essen, Deutschland

**Keywords:** Internationale empirische Forschung, Open Educational Resources, OER, Systematic Mapping, International empirical research, Open Educational Resources, OER, Systematic Mapping

## Abstract

Open Educational Resources (OER) bilden ein wichtiges Element im Diskurs über eine Digitalisierung von Bildung. Der Beitrag erhebt den Stand der internationalen empirischen Forschung zu OER, um dadurch Desiderate für eine zukünftige Forschungsagenda aufzuzeigen. Mittels eines systematischen Mapping-Ansatzes wird dafür die empirische, englischsprachige Forschungslandschaft kartiert. Die Ergebnisse zeigen, dass Forschung vor allem im Hochschulbereich betrieben wird und wenige Studien für den Bereich Schule vorliegen. Forschungsmethodisch sind quantitative Studien prävalent, die vorwiegend umfragebasiert durchgeführt werden. Das primäre Forschungsinteresse der Studien konzentriert sich auf Wahrnehmungsfaktoren von OER sowie auf deren Übernahme und Nutzung in der Bildungspraxis. Ein weiteres Forschungsfeld sind Offene Textbücher und deren komparative Kostenvorteile oder qualitative Vergleichbarkeit mit kostenpflichtigem Bildungsmaterial. Forschungsdesiderate liegen im Feld der Usability respektive Benutzerfreundlichkeit von OER-Repositorien. Forschung hierzu könnte die zahlreichen Bundesländerinitiativen zur Etablierung von OER-Landesportalen unterstützen. Weitere Forschungslücken finden sich bei den Effekten der Nutzung von OER auf das pädagogische Handeln und die Veränderungen etablierter Bildungspraktiken.

## Abstract (en)

Open Educational Resources (OER) are an integral part of the debate about the digitization of education. This article reviews the state of international empirical research on OER to identify possible desiderata for a future research agenda. A systematic mapping approach is used to map the empirical English-speaking research landscape. The results reveal that research concentrates on the higher education sector while only a few studies are available for the school sector. In terms of research methodology, quantitative studies are prevalent, mostly using surveys. The research interest of the studies lies in the perception of OER and their adoption and usage in educational practices. Open Textbooks and their comparative cost advantages or qualitative comparability with traditional educational material is a newly emerging field. Research gaps exist regarding usability and user-friendliness of OER repositories. Addressing these gaps could support the numerous initiatives of the German federal states to establish OER portals. Further research gaps were identified regarding the effects of the use of OER on pedagogical approaches and established educational practices.

## Einleitung

Die Idee der Open Educational Resources (OER), im Deutschen meist als Offene Bildungsmaterialien bezeichnet, bildet ein wichtiges Element im gegenwärtigen Diskurs über eine Digitalisierung von Bildung (vgl. Bozkurt et al. 2019; Zawacki-Richter et al. 2020).

Dabei kann das basale Konzept von OER mittlerweile auf eine fast 20-jährige Geschichte zurückblicken. Geprägt wurde der Begriff erstmals 2002 durch das *UNESCO Forum on the Impact of Open Courseware for Higher Education in Developing Countries* (vgl. UNESCO 2002). Auf zahlreichen internationalen Folgekonferenzen hat sich OER dabei als ein Nukleus der Forderung nach Offener Bildung (Open Education) etabliert (vgl. Zawacki-Richter et al. 2020, S. 321). Diese Forderung kulminierte 2019 in einer zwischenstaatlichen Empfehlung für OER durch die UNESCO-Generalkonferenz (vgl. UNESCO 2019).

Das Kernanliegen von OER ist es, digitale Lehr- und Lernressourcen frei verfügbar und durch die Verwendung möglichst offener Creative Commons-Lizenzen uneingeschränkt nutzbar zu machen (vgl. Wiley und Hilton 2018, S. 134 f.). Wenngleich keine kanonische Definition existiert, beschreibt die letzte von der UNESCO zur Verfügung gestellte Definition OER wie folgt:

learning, teaching and research materials in any format and medium that reside in the public domain or are under copyright that have been released under an open license, that permit no-cost access, reuse, re-purpose, adaptation and redistribution by others (UNESCO 2019, S. 3 f.).

Dieses grundlegende Anliegen von OER ist auch für die Diskussion über die Digitalisierung der deutschen Bildungslandschaft relevant, wobei Deutschland hier im weltweiten Vergleich nur auf den hinteren Plätzen zu verorten ist (vgl. Harwardt 2019). OER wird das Potenzial zugeschrieben, auf ihrer Basis neue didaktische Möglichkeiten auf der Ebene des Lernens, des Unterrichtens und der Zusammenarbeit zu entwickeln und mittels einer offenen Lizensierung den rechtssicheren Einsatz sowie die Weitergabe digitaler Lehr‑/Lernmaterialien zu ermöglichen (vgl. Echterhoff und Kröger 2020). Die Strategie „Bildung in der digitalen Welt“ der Kultusministerkonferenz (KMK) erkannte 2016 das Potenzial von OER an (vgl. Niederastroth 2018). Dieser Anerkennung folgten jedoch nur zaghafte Versuche, OER systematisch in der deutschen Bildungslandschaft zu verankern. Eine erste Bildungsbereich übergreifende nationale Förderinitiative des Bundesministeriums für Bildung und Forschung (BMBF) von 2016 bis 2018 konnte zwar zur Bewusstseinsbildung bezüglich OER beitragen (vgl. Surmann und Echterhoff 2018, S. 20 f.), darüber hinausgehende strukturelle Maßnahmen erfolgen bislang aber eher episodisch denn systematisch (vgl. Otto 2020a, 2021). Somit verbleibt OER in der Diskussion über eine Digitalisierung von Bildung meist auf der strategisch-konzeptionellen Ebene. Dessen ungeachtet existieren vehement Stimmen, die OER gerade aufgrund der neuen Formen des Lehrens und Lernens bedingt durch die Digitalisierung in allen Bildungsbereichen als notwendig ansehen und als ein Vehikel für einen Kulturwandel beschreiben (vgl. Echterhoff und Kröger 2020). Hinweise für diese Notwendigkeit liefern auch jüngere Ereignisse wie die Covid-19-Krise, die den Bildungsbereich übergreifenden Bedarf nach OER aufzeigen. Exemplarisch hierfür ist, dass das BMBF die Schul-Cloud des Hasso-Plattner-Instituts (HPI) für alle Schulen öffnete, um die flächendeckende Nutzung digitaler Lehr- und Lernangebote frei und damit auch zu Hause zu ermöglichen (vgl. BMBF 2020, S. 1 f.).

Wenn also OER von der Bildungspraxis durchaus als wünschenswert erachtet werden, so stellt sich aus bildungswissenschaftlicher Perspektive die Frage des *cui bono*? Konkreter formuliert: Welche potenziellen Wirkungen und möglichen komparativen Mehrwerte gegenüber „traditionellen“ Bildungsmaterialien hat der Einsatz von OER für eine Bildung in einer digitalen Welt?

Wie in Kap. 2 zum deutschsprachigen Forschungsstand aufgezeigt wird, spielen evidenzbasierte Forschungsbefunde in der Diskussion über OER bislang nur eine marginale Rolle. Die Forschung steht damit aber vor dem Problem, dass sie der Bildungspolitik und der Öffentlichkeit in der Debatte über eine Bildung in der digitalen Welt kein belastbares Angebot an Forschungsergebnissen zu OER bereitstellen kann. Ein solches wäre jedoch von eminenter Bedeutung, um OER in der zukünftigen strategischen Ausrichtung und als programmatischen Teil einer Digitalisierungsstrategie verankern zu können.

Da ein deutschsprachiger Forschungsstand nur sehr eingeschränkt vorhanden ist, erhebt der vorliegende Beitrag den Stand der internationalen evidenzbasierten Forschung zu OER. Er systematisiert die bisherigen Befunde und zeigt mögliche Desiderate für eine zukünftige deutschsprachige OER Forschung auf.

## Forschungsstand

Mit Blick auf den aktuellen Stand der deutschsprachigen Forschungsliteratur zu OER zeigt sich ein stark fragmentiertes Bild (vgl. Deimann 2019; Otto 2020a).

Wie auch in anderen Ländern wurde die Entwicklung von OER in Deutschland stark durch die Open-Access-Bewegung beeinflusst und unterstützt deren strategische Forderungen nach einem liberalen, offenen Zugang zu Forschungs- und Bildungsmaterialien (vgl. Mruck et al. 2013). Die Popularität dieser Forderungen manifestiert sich, teilweise initiiert durch die Bildungspolitik, bildungsbereichsübergreifend in Projekten und Initiativen, die frei verwendbare Bildungsressourcen fördern und unterstützen (Deimann 2018).

Die Implikationen von OER für die Bildung werden in der Forschungsliteratur bislang auf verschiedenen Ebenen diskutiert, von denen drei hervorzuheben sind.

Erstens sind hier die pädagogischen und didaktischen Konsequenzen eines Einsatzes von OER in Lehr‑/Lernkontexten zu nennen. Kerres und Heinen (vgl. 2015) unterscheiden zwischen starken und schwachen OER, wobei schwache OER den kostenfreien Zugang und die rechtssichere Verwendung von Lernmaterialien umfassen, starke OER hingegen deren Potenziale für neuartige Lehr‑/Lernszenarien mit digitalen Medien berücksichtigen. Mayrberger und Hofhues (vgl. 2013) fordern, über die reine Fokussierung auf OER hinauszugehen und plädieren dafür, diese in offene Bildungspraktiken im Spektrum von Offenheit, Selbstorganisation(-sfähigkeit) und Partizipation einzubetten.

Ein zweiter Schwerpunkt umfasst die Frage nach einer adäquaten Infrastruktur, um OER verfügbar zu machen. Neben der mangelnden quantitativen Verfügbarkeit von OER stellt deren Auffindbarkeit und effektive Nutzbarkeit eine zentrale Hürde dar. Die Machbarkeitsstudie zum Aufbau und Betrieb von OER-Infrastrukturen von Blees et al. (Blees et al. 2016) kam zu dem Ergebnis, dass eine zentrale bildungsbereichsübergreifende Plattform zur Bündelung aller OER in Form eines einzelnen Repositoriums bzw. Referatoriums weder wünschenswert noch realisierbar erscheint. Zu präferieren sei daher eine Vernetzung bestehender Infrastrukturen und die Entwicklung eines entsprechenden Aggregationsmechanismus, der in der weiteren Diskussion von Kerres et al. (2017) als offene informationelle Ökosysteme konzeptualisiert wurde. Die Idee dahinter besteht darin, Ressourcen unterschiedlicher Granularität und Lizensierung in einer verteilten Bildungsarchitektur zusammenzuführen.

Ein dritter, mit der infrastrukturellen Perspektive eng verbundener, Aspekt ist die Qualitätssicherung von OER. Gerade die den OER inhärente Offenheit und Veränderbarkeit resultiert in der Frage, welche Indikatoren zur Beurteilung von deren Qualität für die Bereiche der Schule und der Hochschule herangezogen werden können und sollen (Brückner 2018). Diese Herausforderung stellt sich besonders mit Blick auf die zunehmende Etablierung von OER-Portalen. Mayrberger und Zawacki-Richter (vgl. 2017) befassten sich in einer ersten Studie mit Instrumenten zur Qualitätssicherung von OER und systematisierten die Ansätze zur Qualitätssicherung und -entwicklung von OER im deutschsprachigen Raum. Basierend auf diesen Ergebnissen entwickelten sie einen umfassenden Vorschlag zur Erstellung eines Qualitätssicherungsinstrumentes am Beispiel der Hamburg Open Online University (vgl. Mayrberger et al. 2018). Hierbei wird zentral zwischen Kriterien der pädagogisch-didaktischen Dimension (Inhalt und didaktische Konzeption) und der technischen Dimension (Zugänglichkeit und Usability) unterschieden.

Obwohl somit Konzepte und Modelle bezüglich OER vorliegen, sind insbesondere evidenzbasierte Forschungsperspektiven nur rudimentär zu erkennen (vgl. Bellinger und Mayrberger 2019; Lechtenbörger 2019; Otto 2019, 2020b). Bildungswissenschaftliche Betrachtungen von OER rekurrieren vorwiegend auf einem reflexiven Forschungszugang, vielfach kulminierend in einer normativen Forderung nach Bildungsgerechtigkeit (vgl. Kerres 2019, S. 2). OER werden dabei eingebettet in eine grundlegendere Forderung nach Offenheit (Openness) im Sinne einer Bildung für Alle (vgl. Peters und Roberts 2011). Hierbei zeigt sich allerdings eine vergleichsweise unreflektierte Verwendung des Bildungsbegriffs, den die Bildungswissenschaft gerade auch im Kontext der Digitalisierung bereits ausdifferenziert hat (vgl. Deimann 2014; Deimann und Farrow 2013).

## Forschungsfragen und Methodik

Für die generische Erschließung eines amorphen Forschungsfeldes ist es essentiell, ein adäquates konzeptionelles Vorgehen zu wählen. Bei dem Konzept der OER kommt erschwerend hinzu, dass dieses über keinen disziplinären Ursprung oder eine originäre Fachdomäne verfügt und sich darüber hinaus keinem spezifischen Bildungsbereich zuordnen lässt (vgl. Otto 2019). Somit sind Forschungsimpulse potenziell aus jedem Fach- oder Bildungsbereich zu erwarten. Um diesen Herausforderungen zu begegnen, verwendet der vorliegende Beitrag den Ansatz des *systematischen Mappings*. Die originäre Idee des systematischen Mapping stammt aus der medizinischen Forschung, wobei die konzeptionelle Umsetzung vor allem im Software Engineering-Bereich etabliert ist (vgl. Fernandez et al. 2011; Petersen et al. 2008), mittlerweile aber auch im Bereich der Bildungswissenschaften erfolgt (vgl. Dicheva et al. 2015; Rasheed et al. 2019).

Untersucht wird bei einem systematischen Mapping die vorhandene wissenschaftliche Literatur zu einem Themenbereich, um einen ersten Überblick über die bisherigen Forschungsbeiträge und besonders die verschiedenen Arten von Forschung zu erhalten, die bislang durchgeführt wurden. Systematische Mapping-Studien bieten so die Möglichkeit, ein Forschungsgebiet zu kartieren, indem Quantität und Qualität, die Arten der Forschung, die verwendete Methodik sowie die bisherigen Schwerpunkte innerhalb des Forschungsbereichs identifiziert werden. Das systematische Mapping kann dadurch Publikationstrends aufzeigen, indem die Häufigkeit von Publikationen im Zeitverlauf abgebildet wird. Das sekundäre Ziel eines systematischen Mappings kann darin bestehen, prävalente Fachdisziplinen und Bildungsbereiche zu identifizieren, in denen die Forschungsergebnisse veröffentlicht werden. Zusammenfassend entsteht damit ein generischer Überblick über das Forschungsgebiet.

In Abgrenzung zum systematischen Mapping untersucht das weit häufiger verwendete systematische Review die relevante verfügbare Forschung in einem etablierten Forschungsgebiet mit dem Ziel, verfügbare Studien dezidiert zu prüfen, bewerten und zu interpretieren (vgl. Newman und Gough, 2020, S. 7 f.). Das systematische Mapping verfolgt dagegen ostentativ das Ziel, ein bislang wenig oder unvollständig erschlossenes Forschungsfeld zu kartieren. Es systematisiert ein sich in der Entstehung befindliches Forschungsfeld in seiner gesamten Breite überblicksartig und eignet sich daher besonders für einen Bereich wie OER, da sich hier zunehmend ein Forschungsfeld etabliert (vgl. Bozkurt et al. 2019; Zawacki-Richter et al. 2020), scheinbar jedoch ein Mangel an bedeutsamen, qualitativ hochwertigen Primärstudien besteht. Übersichtarbeiten wie narrative Reviews (vgl. Clinton 2019) bibliographische Analysen (vgl. King et al. 2018) oder Meta-Analysen (vgl. Otto 2019) liegen nur vereinzelt vor. Der Mehrwert des systematischen Mappings kann hier dementsprechend insbesondere darin bestehen, Desiderate für eine zukünftige Forschungsagenda zu identifizieren.

Um die genannten Ziele zu erreichen, werden anhand der vorliegenden Mapping-Studie zwei Schwerpunktbereiche untersucht. Im ersten Schritt werden auf deskriptiver Ebene die Studiencharakteristika beschrieben. Dafür wurden folgende Forschungsfragen abgeleitet:

- Was sind die bisherigen Schwerpunkte in der OER-Forschung? Lassen sich Prävalenzen bezüglich Fachdisziplinen, Bildungsbereichen und Ländern identifizieren?
- Was sind die forschungsmethodischen Vorgehensweisen bei OER-Studien?

In einem zweiten Schritt werden auf empirischer Ebene die inhaltlichen Schwerpunkte der Studien untersucht, was sich in der dritten Forschungsfrage widerspiegelt:

- Was sind die vorherrschenden thematischen empirischen Schwerpunkte in der OER-Forschung?

## Ablauf des systematischen Mappings

**Figure 1:**
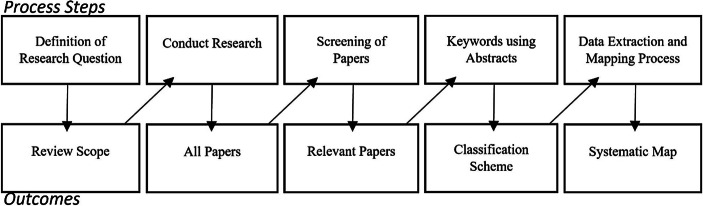

Der Ablauf des systematischen Mappings in diesem Beitrag folgt dem Vorgehen von Petersen et al. (vgl. 2008, S. 2 ff.). Die essentiellen Prozessschritte sind demzufolge die Definition der Forschungsfragen, die Durchführung der Suche nach relevanter Literatur, das Screening der Literatur, die Verschlagwortung der Abstracts sowie die Datenextraktion und -kartierung (vgl. Abb. 1). Das Endergebnis des Gesamtprozesses stellt eine systematische Landkarte dar.

Obwohl das Ziel dieser systematischen Mapping-Studie eine umfassende Darstellung der internationalen empirischen Forschungsliteratur zu OER ist, ergeben sich aufgrund der im Rechercheprozess verwendeten Kriterien verschiedene Limitationen, auf die an den entsprechenden Stellen hingewiesen wird und welche bei der Aussagekraft der Ergebnisse zu berücksichtigen sind.

Für die Recherche wurden die Datenbanken Web of Science, Scopus und ERIC gewählt, die sich für Reviews im Feld der Educational Technology etabliert haben (vgl. Bond et al. 2020; Ramirez-Montoya 2020) und deren Fokus auf peer-reviewed Quellen liegen. Durch die Auswahl der Web of Science Core Collection und Scopus kann eine interdisziplinäre sowie breite geographische Abdeckung sichergestellt werden (vgl. Mongeon und Paul-Hus 2016). Mit dem bildungswissenschaftlichen Schwerpunkt von ERIC konnte darüber hinaus die besondere Berücksichtigung von OER erweitert werden.

Im Vorfeld der Recherche wurden zunächst die Ein- und Ausschlusskriterien für potenzielle Titel festgelegt (vgl. Tab. 1). Um einen möglichst aktuellen Publikationszeitraum abzubilden, wurde Publikationen bis Ende des Jahres 2019 eingeschlossen. 2015 wurde als Beginn gesetzt, da hier die Phase der definitorischen Debatte über OER mit den durch Wiley (vgl. 2014) für OER festgelegten 5 Freiheiten von OER weitestgehend abgeschlossen ist und Wiley et al. (vgl. 2014) zur verstärkten Forschung zu OER aufriefen. Die Einschränkung auf englischsprachige Publikationen erfolgte, um den internationalen Diskurs bestmöglich abzubilden, da dieser für Akteure aus nahezu allen Ländern zugänglich ist. Dementsprechend wurde auf den zusätzlichen Einbezug deutschsprachiger Publikationen aufgrund möglicher Verzerrungen der Ergebnisse verzichtet. Ein weiteres Kriterium betraf den Dokumenttyp. Hier wurden Zeitschriftenartikel ausgewählt, da diese in den ausgewählten Datenbanken den Schwerpunkt bei der Publikationsart ausmachen (vgl. Mongeon und Paul-Hus 2016). Da andere Dokumenttypen, wie Buchkapitel und Konferenzbeiträge, in den Datenbanken nicht umfassend repräsentiert sind, wurden diese ausgeschlossen, auch wenn dadurch möglicherweise bestimmte empirische Studien nicht berücksichtigt wurden.

**Table 1:** 

Kriterium	Einschlusskriterium	Ausschlusskriterium
Publikationsjahr	In den Jahren 2015 bis 2019 veröffentlicht	Vor 2015 oder nach 2019 veröffentlicht
Dokumenttyp	Zeitschriftenartikel	Alle anderen Publikationstypen
Sprache	Englisch	Alle anderen Sprachen
Empirische Studie	Angabe und Anwendung einer Methode für die systematische Analyse der empirischen Daten	Keine Analyse von Daten durchgeführt oder keine Methode für die Analyse der Daten angegeben. → Ausschluss als „nicht-empirisch“
Thematischer Fokus	Open Educational Resources als Schwerpunkt der Studie	Angrenzende Themengebiete ohne Einbezug von OER in Studien → Ausschluss als „nicht-OER“

Mit der Phrase „Open Educational Resources“ erfolgte die Suche bei Web of Science und Scopus über die Suchfelder *Titel, Abstract* und *Keywords*. Bei ERIC erfolgte sie über *alle Felder*, da Abstract dort nicht als einzelnes Suchfeld möglich ist. Entsprechend der aufgeführten Ein- und Ausschlusskriterien *Publikationsjahr, Dokumenttyp* und *Sprache* erfolgte eine Eingrenzung der Treffermengen. Ausgehend von der Prämisse, dass Begriffe in wissenschaftlichen Publikationen im Abstract einmalig ausgeschrieben werden, um diese zu erläutern, wurde bei der Recherche auf das Akronym „OER“ verzichtet. Dadurch wurde eine geringere Trefferquote (Recall) angrenzender Publikationen zugunsten der Genauigkeit (Precision) relevanter Treffer in Kauf genommen (vgl. Ting 2010).

**Figure 2:**
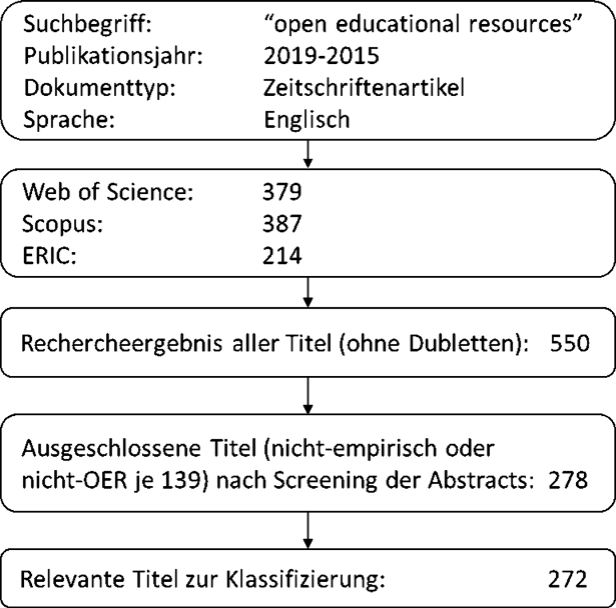

Die am 07.01.2020 durchgeführte Suche ergab nach Reduzierung der Dubletten aus den drei verschiedenen Datenbanken eine Treffermenge von 550 Titeln, die einer inhaltlichen Vorauswahl (Screening) anhand der Abstracts – und wenn notwendig der Volltexte – unterzogen wurden. Dabei wurde geprüft, ob es sich bei den Titeln um empirische Studien handelt und OER den zentralen Untersuchungsgegenstand bildet. So konnten 278 Titel ausgeschlossen werden, bei denen entweder keine empirische Untersuchung durchgeführt oder keine wissenschaftliche Methode zur Datenauswertung expliziert wurde (139 Titel) oder bei denen der thematische Fokus nicht auf OER lag (139 Titel). 272 Titel konnten nach Überprüfung anhand der Kriterien in den finalen Datenkorpus einbezogen werden (Abb. 2).

Auf Grundlage eines von den Autor*innen erarbeiteten und validierten Kategorienschemas konnten die 272 Titel Kategorien zugeordnet werden. Das Kategorienschema wurde nach einem deduktiv-induktiven Verfahren in Anlehnung an Mayring (vgl. 2000) entwickelt, dem folgend die Studien anhand formaler und inhaltlicher Kriterien kategorisiert und analysiert wurden. Die deduktive Bildung der Oberkategorien erfolgte anhand der eingangs aufgestellten Forschungsfragen. Die weiteren Ausprägungen der Oberkategorien in Subkategorien wurden induktiv basierend auf den Inhalten der Publikationen festgelegt. Für dieses Vorgehen diente eine Zufallsstichprobe von 10 % der gefundenen Titel, anhand derer die Kategorien gebildet und diese kommunikativ beurteilt und final validiert wurden. Die inkludierten Studien dienten darüber hinaus dazu, die Intercoderreliabilität zu überprüfen und bei etwaigen Abweichungen Anpassungen vorzunehmen. Die in der Zufallsstichprobe enthaltenen Titel wurde von insgesamt drei Personen kodiert, wobei der Median des Cohens Kappa 0,68 betrug und damit eine substanzielle Übereinstimmung vorlag (vgl. Landis und Koch 1977).

Nach der Finalisierung des Kategorienschemas (vgl. Tab. 2) und der Definition der einzelnen Kategorien erfolgte arbeitsteilig der endgültige Durchgang und die Kategorisierung der 272 Titel^1^.

**Table 2:** 

Kategorie	Subkategorie	
Bildungsbereich	Schule	
	Hochschule	
	Weiterbildung	
	Berufliche Bildung	
	Bildungsbereichsübergreifend	
Forschungsmethodisches Vorgehen	Mixed Methods	
	Quantitativ	Datenanalyse
		Metaanalyse
		Umfrage
		(Quasi‑)Experimentell
		Review
	Qualitativ	Aktionsforschung
		Interview
		Beobachtung
		Fallstudie
		Interpretativer Ansatz
Primärer Fokus	Fokus auf Lernenden	
	Fokus auf Lehrenden	
	Institutioneller Fokus	
	Technischer Fokus	
	Unspezifiziert	
Thematische Forschungsschwerpunkte	Infrastructure	
	Usage, Adoption	
	Perception, Attitude	
	Learning outcomes	
	Effectiveness OER	
	Barriers	
	Quality of Material	
	Strategy	
Verwandte Konzepte	Open Science	
	MOOCs	
	Open Textbooks	
	Open Educational Practices	
	Open Pedagogy	
	Open Access	
Originärer Beitrag	Empfehlungen	
	Evaluation/Lessons Learned	
	Theoriebildung	
	Lehrstrategien	

Auf der formalen Ebene umfasste das Kategorienschema *Bildungsbereiche* (*Schule, Hochschule, Weiterbildung, berufliche Bildung*), *Fachbereiche* sowie *Länder* bzw. *Kontinente*, in den die Studien durchgeführt wurden.

Mit der Kategorie des *forschungsmethodischen Vorgehens* werden die in den Studien eingesetzten Methoden analysiert, wobei zwischen *quantitativen Methoden* (*Datenanalyse, Umfrage, Experiment, Metaanalyse* sowie *Reviews*), *qualitativen Methoden* (*Interviews, Beobachtung, Fallstudien* und *interpretative Ansätze*) sowie *Mixed Methods* unterschieden wurde.

Auf der inhaltlichen Ebene besteht eine Kategorie aus dem *primären Fokus* der Untersuchung. Bei verschiedenen Ausprägungen dieser Kategorie handelt es sich um die praktische Anwendung von OER in einem Lehr‑/Lernkontext (*Fokus auf Lernenden*) oder die Erstellung und Nutzung von OER durch Lehrende (*Fokus auf Lehrenden*). Daneben kann der Fokus auch auf der institutionellen Ebene (*Institutioneller Fokus*) liegen, wobei es sich hier eher um strategische Implementationen von OER, eine Kultur des Teilens oder die Potenziale und Grenzen von OER auf Makro-Ebene handelt. Ein Fokus auf technischen Aspekten (*Technischer Fokus*) liegt vor, wenn beispielsweise die Beschaffenheit von OER-Repositorien oder die Auffindbarkeit von OER analysiert werden oder es um das Testen neuer Services oder Tools im Bereich OER geht.

In Abgrenzung zum primären Fokus der Untersuchung werden anhand der Kategorie *Thematische Forschungsschwerpunkte* die inhaltlichen Dimensionen der Studien genauer ausdifferenziert. Dabei wird davon ausgegangen, dass Studien mehrere inhaltliche Dimensionen enthalten können, so dass ein Artikel, im Gegensatz zu allen anderen Kategorien, mehrfach zugeordnet werden konnte. Bei den thematischen Forschungsschwerpunkten ergaben sich die folgenden inhaltlichen Differenzierungen^2^: Technische Infrastruktur (*Technical Infrastructure*); Nutzung von OER (*Usage, Adoption*) mit Erstellung, Anpassung und Teilen von Materialien; Wahrnehmung und Haltung zu OER (*Perception, Attitude*); Ergebnisse bei dem Einsatz von OER in Lernsettings (*Learning Outcomes*); Auswirkungen von OER auf Kosten, Zeit oder Zusammenarbeit *(Effectiveness*); Hindernisse (Barriers) sowie Anreize für OER (*Incentives, Motivation*); Qualität von Materialien (*Quality*) und strategische Aspekte von OER (*Strategy*).

Weiterhin wurden auch mit OER *verwandte Konzepte* erhoben, die in den OER-Studien Gegenstand der Untersuchung waren. Bei diesen Konzepten handelte es sich unter anderem um *Open Textbooks, Massive Open Online Courses* (MOOCs), *Open Educational Practices *oder *Open Science*.

Die letzte Kategorie der inhaltlichen Aspekte bildete der *originäre Beitrag* der jeweiligen Studie. Subkategorien waren hier eine rückblickende *Evaluation* von Maßnahmen; Entwicklung von *Empfehlungen*; *Lehrstrategien* oder *Theoriebildung*.

## Ergebnisse

Die folgende Auswertung des systematischen Mappings erfolgte auf Basis der quantitativen Verteilung der Studien auf die einzelnen Kategorien, auf Grundlage derer systematische Darstellungen anhand der eingangs definierten Forschungsfragen abgeleitet werden konnten.

Bei den 272 Titeln, die in die Auswertung eingeflossen sind, lässt sich zur Verteilung auf die Erscheinungsjahre 2015 bis 2019 Folgendes festhalten: Während aus dem Jahr 2015 die geringste Anzahl empirischer Studien in die Untersuchung eingeflossen sind, markiert das Jahr 2017 mit 69 Titeln die höchste Anzahl untersuchter Titel. Nach einer geringfügigen Reduzierung im Jahr 2018 (51 Titel) steigt die Anzahl der kartierten Publikationen im Jahr 2019 wieder auf ein Niveau von 65 Titeln.

Bei der Verteilung der Studien nach den Ländern, wobei diese sich auf die Teilnehmer*innen der Studien, nicht auf die jeweiligen Autor*innen beziehen, sind diese der Übersichtlichkeit halber auf die Ebene des jeweiligen Kontinents kumuliert worden (vgl. Tab. 3).

**Table 3:** 

Kontinent	Nordamerika	Europa	Asien	Übergreifend	Afrika	Südamerika	Nicht spezifiziert	Australien/Ozeanien
Anzahl	101	53	47	20	19	10	14	8
Prozent	37,13	19,49	17,23	7,35	6,99	3,68	5,15	2,94

Für 14 Publikationen konnte keine eindeutige Zuordnung ausgemacht werden, während 20 Erhebungen auf mehr als einem Kontinent durchgeführt wurden. Bei der Verteilung der Studien auf die Kontinente zeigt sich, dass mit ca. 37 % mehr als ein Drittel der Studien in Nordamerika zu verorten sind und ein Großteil dieser Studien aus den USA stammt (vgl. Tab. 4, in der die Länder, die in Tab. 3 kumuliert sind, detailliert aufgelistet werden). Es folgen Studien aus Europa und Asien mit etwa 19 % bzw. 17 %. Kaum ins Gewicht fallen dagegen mit ca. 7 % bzw. 4 % Studien aus Afrika und Lateinamerika, wobei gerade für diese Regionen häufig ein Bedarf nach OER attestiert wird (vgl. Lambert 2018).

**Table 4:** 

Übergreifend	Nordamerika	Europa	Asien	Afrika	Südamerika	Australien/Ozeanien
Unspezifiziert (17) Korea + Japan + USA (1) Spanien + Griechenland + Australien (1)	USA (86) Kanada (8) Mexiko (6) Kanada + USA (1)	Vereinigtes Königreich (17) Spanien (8) Griechenland (4) Deutschland (4) Niederlande (3) Kroatien (2) Portugal (2) Schweden (2) Irland (1) Italien (1) Estland (1) Frankreich (1) Litauen (1) Luxemburg + Montenegro + Deutschland + Irland (1) Norwegen (1) Spanien + Vereinigtes Königreich (1) Ungarn (1) Unspezifiziert (2)	Indien (10) China (8) Indonesien (4) Sri Lanka (4) Taiwan (4) Türkei (3) Israel (3) Japan (1) Japan + Malaysia (1) Jordanien (1) Korea (3) Kirgistan (1) Kasachstan (1) Philippinen (1) Thailand (1) Saudi-Arabien (1)	Südafrika (7) Sambia (3) Kenia (2) Nigeria (2) Ghana (1) Kamerun (1) Ruanda (1) Unspezifiziert (2)	Brasilien (3) Unspezifiziert (3) Kolumbien (2) Chile (1) Venezuela (1)	Australien (6) Neuseeland (1) Fidschi (1)

Bei Betrachtung der Bildungssektoren wird deutlich, dass empirische Forschung größtenteils den Hochschulbereich fokussiert (vgl. Tab. 5): Dem Bildungsbereich Hochschule lassen sich etwa 71 % der untersuchten Studien zuordnen, während nur knapp 14 % aus dem Bereich Schule stammen und andere Bildungsbereiche, wie Berufsbildung (>1 %) und Weiterbildung (ca. 2,6 %), sich eher auf einem marginalen Niveau bewegen. Bildungsbereich übergreifende Forschung konnte für etwa 13 % der Studien erfasst werden und spielt im Bereich der empirischen OER-Forschung eine nicht zu vernachlässigende Rolle. Zugeordnet sind dieser Kategorie Bildungsbereich übergreifende Reviews oder Studien, welche übergreifende Themen behandeln, die sich nicht auf einen konkreten Bildungsbereich beziehen. Dies umfasst auch Studien, die bspw. die Nutzung von Repositorien untersuchen und dabei keine Daten zum Bildungsbereich erheben, weil lediglich Nutzer*innen betrachtet werden.

**Table 5:** 

Bildungsbereich	Hochschule	Schule	Bildungsbereich-übergreifend	Weiterbildung	Berufliche Bildung
Anzahl	192	37	34	7	2
%	70,59	13,6	12,5	2,57	0,74

Die folgende Zuordnung der Studien zu Fachbereichen bzw. Disziplinen, die im Zentrum der jeweiligen Untersuchungen standen, basiert auf der obersten Ebene der Fachsystematik der Deutschen Forschungsgemeinschaft (DFG 2020). Die Ergebnisse zeigen deutlich, dass fächer- bzw. disziplinübergreifende Studien und solche, in denen die untersuchten Fachbereiche von den Autor*innen nicht näher spezifiziert wurden, mit fast 56 % mehr als die Hälfte der Studien ausmachen. Es folgen die Geistes- und Sozialwissenschaften mit knapp 24 % und die Naturwissenschaften mit ca. 12 %. Den sog. Lebenswissenschaften konnten etwa 5 %, den Ingenieurswissenschaften fast 4 % aller Studien zugeordnet werden (vgl. Tab. 6).

**Table 6:** 

Fachdisziplin	>1 oder nicht spezifiziert	Geistes- und Sozialwiss.	Naturwissenschaften	Lebenswissenschaften	Ingenieurswissenschaften
Anzahl	152	65	32	13	10
%	55,88	23,9	11,76	4,78	3,78

Die in den Studien angewendeten Forschungsmethoden lassen bereits auf der obersten Ebene erkennen, dass quantitative Methoden mit knapp 63 % prävalent sind, während rein qualitative Methoden mit gut 20 % einen recht geringen Anteil ausmachen und sich auf einem ähnlichen Niveau bewegen wie Mixed-Method-Ansätze mit einem Anteil von etwas mehr als 17 % (vgl. Tab. 7). Werden die drei Erhebungsarten im Detail betrachtet, so zeigt sich, dass im quantitativen Bereich in erster Linie Umfragen und Datenanalysen (Deskriptiv- und Korrelationsanalysen, experimentelle und quasi-experimentelle Designs, Data Mining, Learning Analytics) durchgeführt werden und in den qualitativen Studien Interviews und interpretative Studien vorherrschen.

**Table 7:** 

	Forschungsmethode	Anzahl	%
Quantitativ (62,5 %)	Umfrage	88	31,6
	Datenanalyse	54	19,9
	Reviews	15	5,5
	(Quasi‑) Experimentell	12	4,4
	Meta-Analyse	4	1,1
Qualitativ (20,3 %)	Interview	20	7,4
	Interpretative Ansätze	19	7,0
	Fallstudie	13	4,8
	Aktionsforschung	2	0,7
	Beobachtung	1	0,4
Mixed Methods (17,2 %)		47	17,2

Im Anschluss an die Analyse auf formaler Ebene wurden auf der inhaltlichen Ebene die primären Untersuchungsfokusse und thematische Forschungsschwerpunkte der Studien ermittelt. Bei dem primären Untersuchungsfokus wurde jeder Studie jeweils nur der dominierende Fokus zugeordnet, was jedoch nicht ausschließt, dass innerhalb einer Studie weitere Untersuchungsfokusse vorhanden sein können, die jedoch durch die einmalige Kodierung jeder Studie nicht erhoben wurden.

Bei der Kategorisierung der Studien nach dem primärem Untersuchungsfokus (vgl. Tab. 8) lässt sich feststellen, dass die Untersuchung von Lernenden mit knapp 39 % am häufigsten im Zentrum steht, gefolgt von Lehrenden/Kursleitenden mit 33 %. Nur etwa 14 % der Studien konnten der institutionellen Ebene zugeordnet werden, während technische Aspekte lediglich bei 12 % der Studien als primärer Untersuchungsfokus festgestellt wurden.

**Table 8:** 

Primärer Untersuchungsfokus	Fokus auf Lernenden	Fokus auf Lehrenden	Institutioneller Fokus	Technischer Fokus	Unspezifiziert
Anzahl	106	90	37	33	6
%	38,97	33,09	13,60	12,13	2,21

**Figure 3:**
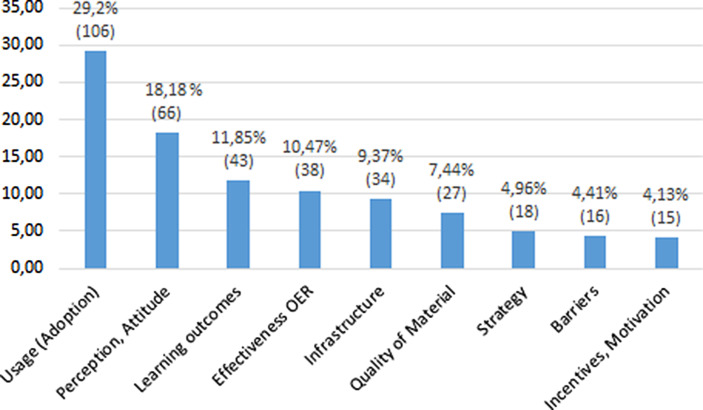

Anhand der thematischen Forschungsschwerpunkten erfolgte eine inhaltliche Ausdifferenzierung der Studien (vgl. Abb. 3). Hier war eine Zuordnung der Studien zu mehreren Subkategorien möglich, um die gesamte Bandbreite der Themen der Studien abdecken zu können. Obwohl das systematische Mapping keine detaillierte inhaltliche Analyse der einzelnen Studien vorsieht, können so die einzelnen thematischen Forschungsschwerpunkte, neben der Darstellung der quantitativen Verteilung der Studien, zumindest kursorisch illustriert werden.

Auffällig ist bei der Betrachtung der thematischen Forschungsschwerpunkte (vgl. Abb. 3), dass fast 30 % der Studien der Kategorie *Usage, Adoption* zugeordnet werden konnten. Hier sind einerseits Studien zu verorten, bei denen die Erstellung und Nutzung von OER durch Lehrende, selten von Lernenden, erhoben und untersucht sowie klassifiziert werden, bspw. anhand des OER-Lifecycles (vgl. Schuwer und Janssen 2018) oder der OER-Pyramide (Baas et al. 2019). Andererseits präsentieren verschiedene Studien Erfahrungsberichte (Evaluation, Lessons Learned), von der Erstellung und Nutzung offener Bildungsressourcen in Lernsettings (vgl. Hassan et al. 2019), oder sprechen erfahrungsbasierte Empfehlungen aus, wie diese integriert werden können (vgl. Sandanayake 2019).

Bei Studien, die sich auf Lehrende/Kursleitende oder Lernende konzentrieren, wurden neben der Erstellung und der Nutzung von OER häufig auch Einstellungen zu oder die Wahrnehmung von OER erhoben. Entsprechende Studien wurden der Kategorie *Perception, Attitude* zugeordnet, was auf etwa 18 % aller untersuchten Studien zutraf. Dies umfasst einerseits Studien, die auf die Untersuchung der allgemeinen Wahrnehmung von und der Einstellungen zu OER abzielen, bspw. hinsichtlich der damit verbundenen Möglichkeiten oder Herausforderungen sowie der Bereitschaft, Ressourcen zu teilen (vgl. Ozdemir und Bonk 2017). Andererseits wurden dieser Kategorie Studien zugeordnet, in welchen die Wahrnehmung von und die Einstellung zu OER erhoben wurden, nachdem diese in einem konkreten Lernsetting genutzt und dabei ggf. geschlossene Ressourcen ersetzt haben (vgl. Illowsky et al. 2016).

Bei Studien, die einen Fokus auf die Lernenden legten, wurde darüber hinaus oft ein Fokus auf die Evaluation von mit OER erzielten Lernergebnissen gelegt. Insgesamt konnten knapp 12 % der Studien der entsprechenden Kategorie *Learning Outcomes* zugeordnet werden. In diesen Studien wurde, z. B. in experimentellen Settings oder mit Hilfe von Umfragen oder Learning Analytics, verglichen, inwieweit sich Lernergebnisse mit geschlossenen Bildungsmaterialien von jenen mit offenen Materialien unterscheiden (vgl. Hilton 2019). Viele der Studien deuten darauf hin, dass die Nutzung offener versus geschlossener Materialien keinen signifikanten Einfluss auf den Lernerfolg (Noten, Abschlussquote, Abbruchquote) hat. Der Forschungsstand indiziert demnach, dass offene Bildungsmaterialien durchaus ein adäquates Substitut für geschlossene Angebote darstellen, mit denen Kosteneinsparungen möglich sind und die Passung von Lernmaterialien und -inhalten verbessert werden kann (vgl. Cuttler 2019).

In diesem Zusammenhang ist auch die Betrachtung der Kategorie *Effectiveness* interessant, welcher 11 % der Studien zugeordnet werden konnten. Hierbei handelt sich jedoch nicht um die Effektivität in Bezug auf Lernergebnisse, sondern um Aspekte wie Kosten- oder Zeiteffizienz, auch im Vergleich zu geschlossenen Materialien. Viele Studien dieser Kategorie evaluieren offene Bildungsmaterialien hinsichtlich ihrer Qualität und Eignung für Lehr‑/Lernkontexte. In diesem Zusammenhang sind diverse Studien insbesondere aus dem US-Amerikanischen Raum hervorzuheben, welche den Einsatz offener Textbücher und ihre komparativen Kostenvorteile gegenüber konventionellen Bildungsmaterialien untersuchen. Dieser Forschungsschwerpunkt scheint nachvollziehbar, da OER in vielen Ländern eingesetzt werden, um die Kosten für Bildung zu reduzieren. In den USA können solche Studien vor allem im universitären Kontext verortet werden. Meist werden dabei die Qualität und die Akzeptanz offener Kursmaterialien mit denen geschlossener Kursmaterialien verglichen und die Substitution kostenpflichtigen Materials durch offene Äquivalente evaluiert. 17 der betrachteten 272 Studien wiesen einen solchen Fokus aus, was auch im Rahmen der Analyse der mit OER verwandten Konzepte (vgl. Tab. 9) deutlich wurde. Hierbei wurde untersucht, ob thematisch mit OER verwandte Begriffe in den begutachteten Studien eine Rolle spielten. Auffallend ist in diesem Zusammenhang zunächst, dass Themen wie Open Pedagogy oder Open Educational Practices (OEP) nur in jeweils drei resp. vier Studien eine Rolle spielten. Allerdings ist das geringe Vorhandensein dieser Konzepte in den untersuchten Studien möglicherweise auch auf die Beschränkung der Suche nach „Open Educational Resources“ zurückzuführen.

**Table 9:** 

Verwandte Konzepte	MOOCs	Open Textbook	Open Access	Open Pedagogy	Open Educational Practices	Open Science
Anzahl	2	17	2	3	4	0

Das auch in der deutschsprachigen Forschung diskutierte Thema der Qualität von Materialien (vgl. Kap. 2) findet sich in 7 % aller Studien wieder. Ein Strang der dieser Kategorie zugeordneten Studien thematisiert die Bewertung offener Materialien anhand verschiedener Kriterien wie Relevanz; Ästhetik, Verständlichkeit; Inhaltliche Qualität (vgl. Cuttler 2019). Ein weiterer Strang thematisiert den Prozess der Qualitätssicherung bei der (z. T. kollaborativen) Erstellung und Evaluation von offenen Bildungsressourcen (vgl. Marín et al. 2019), während ein dritter Strang die Analysekriterien zur Bewertung von OER selbst fokussiert (vgl. Yuan und Recker 2015).

Darüber hinaus ist auffallend, dass bei den Studien, die den genannten Forschungsschwerpunkten zugeordnet wurden, meist Lehrende oder Lernende untersucht wurden, während andere Akteure, z. B. in Bibliotheken oder der Verwaltung, kaum untersucht werden. Dies ändert sich bei der Betrachtung der Kategorien *Infrastructure* oder *Strategy*, denen knapp 10 % resp. 5 % der Studien zugeordnet werden konnten. Hierbei geht es nicht primär darum, wie OER erstellt, genutzt, wahrgenommen oder evaluiert werden bzw. welche Effekte mit ihrer Nutzung erzielt werden können. Vielmehr wird untersucht, wie auf struktureller Ebene die Voraussetzungen für die Nutzung von OER geschaffen bzw. verbessert oder wie Implementationsstrategien oder Policies erarbeitet oder umgesetzt werden können. Dabei spielen neben Lernenden und Lehrenden dann auch weitere Akteure, z. B. der technische Support, Bibliotheken oder nicht-lehrendes Personal, eine Rolle.

Der Kategorie *Infrastructure* wurden einerseits Studien zugeordnet, in denen es um Umgebungen, Systeme, Methoden oder Tools geht, die das Auffinden und Kuratieren oder das Erstellen, Nutzen, Remixen und Teilen von OER ermöglichen oder verbessern (vgl. Moundridou et al. 2019). Andererseits wurden der Kategorie *Infrastructure* Studien zugeordnet, die die Rolle von Bibliotheksangestellten bei der Unterstützung von Fakultäten bei der Nutzung von OER untersuchen (vgl. Braddlee und Vanscoy 2019).

Die Kategorie *Strategy* enthält Studien, die sich bspw. damit beschäftigen, welche Faktoren die flächendeckende Nutzung oder Verbreitung von OER aus der Perspektive einer Institution (Marko-Ebene) beeinflussen (vgl. Masterman 2016) oder wie OER-Aktivitäten unter Berücksichtigung verschiedener Aspekte, wie der institutionellen Kultur, übergreifender Strategien und Policies sowie vorherrschender Geschäftsmodelle initiiert und ausgeweitet werden können (vgl. Jung et al. 2017).

In diesem Kontext ist auch auf die Hindernisse hinzuweisen, die die Nutzung offener Bildungsressourcen unterminieren. Etwa 4,5 % der untersuchten Studien beschäftigen sich mit dieser Thematik und können somit der Kategorie *Barriers* zugeordnet werden. Die in den Studien genannten Hindernisse sind äußerst heterogen und reichen von fehlenden Policies oder Anreizsystemen seitens der jeweiligen Bildungsinstitution und fehlender technologischer Infrastruktur über ein fehlendes Bewusstsein für OER bis hin zu einer schweren Auffindbarkeit von Materialien, der fehlenden Passung dieser und fehlendem Wissen bzw. mangelnden Kompetenzen (vgl. Henderson und Ostashewski 2018).

Knapp über 4 % der untersuchten Studien beschäftigen sich analog dazu mit motivationalen und weiteren Faktoren, die die Nutzung von OER beeinflussen, und wurden somit der Kategorie *Incentives, Motivation* zugeordnet (vgl. Belikov und Bodily 2016).

Interessante Ergebnisse ergeben sich bei der Betrachtung der Kategorie des originären Beitrags in Tab. 10. Diese zeigt deutlich, dass sich mit fast 80 % ein Großteil der Studien eine Evaluation durchgeführter Projekte, Maßnahmen oder Intervention fokussiert. Kaum vorhanden sind dagegen Beiträge, die konkrete (Handlungs‑) Empfehlungen für den Umgang mit OER entwickeln. Ebenfalls kaum verfügbar sind Beiträge zur Theoriebildung oder Ausarbeitung von Lehr- oder Implementationsstrategien. Dies lässt sich als Indiz dafür deuten, dass sich die empirische Forschung zu OER noch in ihren Anfängen befindet und sich scheinbar noch keine neuen und bestehenden Theorien oder Modelle für OER etabliert haben. Gerade deshalb bildet dieses bislang wenig beachtete Feld – zumindest im Kontext bildungswissenschaftlicher empirischer Beiträge – die Möglichkeit, Theorien des pädagogischen Handelns und Lehrkonzepte verstärkt in den Fokus zu nehmen (vgl. Wiley und Hilton 2018).

**Table 10:** 

Originärer Beitrag	Evaluation/Lessons Learned	Empfehlungen	Theoriebildung	Lehrstrategien
Anzahl	210	44	15	3
%	77,21	16,18	5,51	1,1

## Diskussion

In dem vorliegenden Beitrag wurde die internationale englischsprachige empirische Forschungsliteratur zum Thema OER unter Verwendung eines systematischen Mapping-Ansatzes erhoben.

Die Ergebnisse lassen prima facie den Schluss zu, dass es sich bei OER um ein Forschungsfeld handelt, dass sich in empirischer Hinsicht in einem Entstehungsprozess befindet. Darauf deuten die insgesamt 272 Studien hin, welche die in Tab. 1 aufgeführten Einschlusskriterien erfüllten und in die Untersuchung einbezogen werden konnten.

Für die eingangs aufgeworfenen Forschungsfragen lassen sich folgende Befunde ableiten:

### Was sind die bisherigen Schwerpunkte in der OER-Forschung? Lassen sich Prävalenzen bezüglich Fachdisziplinen, Bildungsbereichen und Ländern identifizieren?

Hinsichtlich der Länderverteilung zeigt sich, dass Forschung zu OER primär im Nordamerikanischen Raum betrieben wird (vgl. Tab. 3 und 4). Mit wesentlichem Abstand folgen der europäische und der asiatische Raum. Auffällig ist, dass wenige Studie für Regionen vorliegen, die häufig in der Literatur als die potenziell größten Profiteure von OER angesehen werden, nämlich die Länder des globalen Südens (vgl. Kerres 2019; King et al. 2018).

Bei der Verteilung nach Bildungsbereichen liegt der Fokus eindeutig auf der Hochschule, gefolgt von der Schule (vgl. Tab. 5). Es zeigt sich weiterhin, dass ein nicht unerheblicher Anteil der Studien Bildungsbereich übergreifend durchgeführt werden. Dies bietet sich gerade bei Themenstellungen wie OER-Repositorien an oder, wenn die Akzeptanz und die Wahrnehmung von OER anhand von Umfragen gemessen werden.

Dass es sich bei OER um ein Themenfeld handelt, das nicht nur keinem Bildungsbereich, sondern auch keiner Fachdisziplin eindeutig zugeordnet werden kann, illustriert die Zuordnung der Studien zu Fachdisziplinen (vgl. Tab. 6). Über die Hälfte aller Studien werden in mehr als einer Fachdisziplin durchgeführt. Es lassen sich daher keine Auffälligkeiten von OER hinsichtlich bestimmter Fachbereiche attestieren.

### Welches sind die forschungsmethodischen Vorgehensweisen bei OER-Studien?

Hier zeigt sich ein eindeutiger Schwerpunkt bei quantitativen Studien, insbesondere Umfragestudien (vgl. Tab. 7). Dies resultiert vor allem daraus, dass ein eminenter Forschungsschwerpunkt der Studien auf der Untersuchung der Wahrnehmung und Akzeptanz von OER durch verschiedene Akteursgruppen liegt und bspw. erhebt, ob Lehrende OER kennen oder wie diese OER einschätzen und bewerten. Datenanalysen bilden einen zweiten Schwerpunkt. Dieser umfasst z. B. die Untersuchung von OER-Repositorien oder die Beschaffenheit und quantitative Verfügbarkeit von OER, was neben der mangelnden Bekanntheit eines der großen Probleme bei der Verbreitung von OER darstellt.

Im Gegensatz zu den quantitativen Methoden sind Studien, die qualitative Methoden verwenden, zu einem deutlich geringeren Anteil vorhanden. Vor dem Hintergrund, dass Studien zur Nutzung von OER thematisch am häufigsten vertreten sind, würden sich qualitative Vorgehensweisen anbieten, um die Erstellung und Anpassung von Materialien in der Praxis von Lehrenden im Detail zu untersuchen.

Dass es sich bei der bisherigen OER-Forschung um ein Forschungsfeld im Entstehungsprozess handelt, zeigt sich in methodischer Hinsicht in der geringen Verfügbarkeit von Meta-Analysen oder Reviews. Ein zentrales Problem hierfür könnte die generell geringe Verfügbarkeit von hochwertigen Studien sein, insbesondere zu spezifischen Fragestellungen/Themenbereichen.

### Was sind die vorherrschenden thematischen empirischen Schwerpunkte in der OER-Forschung?

Bei den Untersuchungsfokussen der Studien lässt sich attestieren, dass sich diese eindeutig auf Lernende oder Lehrende richten (vgl. Tab. 8). Dabei wird überwiegend darauf abgezielt, die Übernahme von OER sowie Wahrnehmungen und Erfahrungen mit diesen durch beide Gruppen zu untersuchen. Bemerkenswert ist, dass OER meist kontrastierend mit geschlossenen Bildungsmaterialien untersucht werden und nicht als alleiniger Untersuchungsgegenstand. Letzterer Typ von Untersuchungen basiert häufig auf Einzelfallstudien oder Erfahrungsberichten von Lehrenden. Unterrepräsentiert sind dagegen Studien, die institutionelle oder technische Bedingungen für OER beleuchten. Dies kann auch durch andere Befunde und Berichte bestätigt werden, die feststellen, dass bisher in kaum einem Land adäquate politische oder strukturelle Bedingungen für den Einsatz von OER existieren (vgl. Orr et al. 2017; UNESCO IITE 2019). Insgesamt trägt dies auch dazu bei, dass Folgefragen nach den technischen und infrastrukturellen Standards für den Aufbau und die Ausgestaltung von OER-Repositorien noch nicht abschließend geklärt sind (Atenas und Havemann 2014; Clements et al. 2015).

Diese erkennbaren Tendenzen bei den Untersuchungsfokussen werden durch die Ergebnisse der ausdifferenzierteren thematischen Forschungsschwerpunkte weiter erhärtet (vgl. Abb. 3). Von wenigen empirischen Studien adressiert wird bisher die Entwicklung von institutionellen Strategien (vgl. Bossu und Stagg 2018) sowie die technische Infrastruktur (vgl. Clements et al. 2015; Cohen et al. 2015). Die dominierende Anzahl von Studien konzentriert sich auf Faktoren, welche im unmittelbaren Zusammenhang mit den OER stehen: Wie werden OER wahrgenommen oder genutzt? Lassen sich mit OER ähnliche Lernergebnisse erzielen wie mit traditionellen Materialien und sind diese ebenso effektiv? Letzterer Aspekt manifestiert sich bei den OER-Studien und den darin auftauchenden verwandten Konzepten in zahlreichen Untersuchungen zum Einsatz Offener Textbücher (vgl. Tab. 9). Diese Studien stammen ausschließlich aus den USA, wo die Idee von auf OER-basierenden Lehrbüchern viel Beachtung findet, besonders vor dem Hintergrund der im Vergleich zu Deutschland exorbitanten Kosten von Bildungsmaterialien (vgl. Clinton 2019; Hilton 2016). Inwiefern der Einsatz von Offenen Textbüchern auch für den deutschsprachigen Raum interessant ist, könnte ein Aspekt einer zukünftigen OER-Forschungsagenda sein. Vergleichsweise unterrepräsentiert bei den verwandten Konzepten sind jene, die sich auf Basis von OER entwickelt haben und bislang zumindest auf konzeptioneller Ebene rege diskutiert werden. Dennoch erfahren empirisch weder die Idee von Offenen Bildungspraktiken (OEP), verstanden als Veränderungseffekte, die durch den Einsatz von OER auf etablierte individuelle und institutionelle Praktiken entstehen (vgl. Cronin und MacLaren 2018), noch das von einigen Autor*innen aus den spezifischen Charakteristika der OER abgeleitete Konzept der Offenen Pädagogik (vgl. Wiley und Hilton 2018) in den Studien eine bedeutende Umsetzung. Nur eingeschränkt gültig ist dieser Befund, da durch die verwendete Methodik der Mapping-Studie mögliche empirische Artikel über OEP und Offenen Pädagogik, die keinen expliziten Bezug zu OER nehmen, ausschlossen sind. Für beide Konzepte existiert in der Forschungsliteratur jeweils ein weites und ein enges Verständnis, wobei nur letzteres die explizite Anwendung von OER vorsieht (vgl. Bellinger und Mayrberger 2019; Cronin und MacLaren 2018; Wiley und Hilton 2018). Jedoch monieren auch jüngste Studien, zumindest für die Offene Pädagogik, dass hier kaum empirische Befunde vorliegen (vgl. Hilton et al. 2020).

Abschließend bestätigt auch der Aspekt des primären Beitrags der Studien (vgl. Tab. 10) den Eindruck, dass sich die bisherige Forschung zu OER in erster Linie darauf konzentriert, den basalen Fundus an verfügbaren empirischen Studien zu verbreitern, in dem individuelle oder institutionelle Maßnahmen beispielsweise an Hochschulen oder im Rahmen von Kursangeboten evaluiert und berichtet werden. Das gerade im Bereich der Mediendidaktik/Bildungstechnologie häufig monierte Fehlen von theoriegeleitetem Vorgehen und/oder Beiträgen, die zur Theoriebildung beitragen (vgl. Hew et al. 2019), lässt sich auch für das Forschungsfeld OER bestätigen. Ein Forschungsdesiderat, wie in Kap. 5 ausgeführt, wäre es daher, zu prüfen, inwiefern sich etablierte Ansätze und Theorien der Bildungswissenschaft eignen, um die empirische OER-Forschung theoretisch zu fundieren und damit auch zu systematisieren. Unter anderem auf dieser Basis könnte ein weiterer unterrepräsentierter Aspekt, nämlich konkrete Handlungsempfehlungen, genauer eruiert werden.

Hier bieten sich besonders „Theorien mittlerer Reichweite“ (vgl. Merton 1964) an, die sowohl empirische Befunde konkreter erklären können, als auch das Potenzial besitzen, ein Forschungsdesign zu konzeptualisieren und darauf basierend die Ergebnisse zu interpretieren. Für OER scheinen Theorien über die Integration von digitalen Technologien geeignet, beispielsweise das Technologieakzeptanzmodell (TAM) (vgl. Davis et al. 1989) das Technological Pedagogical Content Knowledge (TPACK) Modell (vgl. Koehler und Mishra 2008) oder die Selbstbestimmungstheorie (vgl. Deci und Ryan 1993). Darüber könnten möglicherweise bestehende Ansätze zur Lehrerkooperation (vgl. Fussangel und Gräsel 2012) für OER Studien interessant sein.

## Ausblick

Zusammenfassend liegen Forschungsdesiderate, die gerade auch für eine deutschsprachige OER-Forschungsagenda vielversprechend sein könnten, in der bildungstheoretischen Fundierung vieler nicht stringenter Studiendesigns. Dies kann zu einer Systematisierung und Validierung der Aussagekraft der verschiedenen Studien beitragen und auch die häufig nur induktiv geäußerte Einflussvariablen identifizieren. Noch intensivere Forschung zur Usability respektive Benutzerfreundlichkeit von OER-Repositorien, in denen OER zu Verfügung gestellt und von Nutzer*innen hoch- oder heruntergeladen werden (vgl. Atenas und Havemann 2014; Clements et al. 2015; Cohen et al. 2015), könnte dazu beitragen, die zahlreichen Bundesländerinitiativen zur Etablierung von Landesportalen für OER unterstützen. Weitere Forschungslücken finden sich bei den wenig untersuchten empirischen Effekten der Nutzung von OER auf das pädagogische Handeln und die möglichen Veränderungen von etablierten Bildungspraktiken, zu denen kaum Studien vorliegen.

Die systematische Mapping-Studie liefert somit einen ersten wichtigen Impuls für eine zukünftige deutschsprachige OER-Forschungsagenda, mit der das Potenzial von OER als einem Teilaspekt für eine Teilhabe an einer Bildung in der digitalen Welt evidenzbasiert bestimmt werden kann.

## Footnotes

1 Zur besseren Nachvollziehbarkeit sind jeweiligen Kategorien und Subkategorien in der folgenden Zusammenschau kursiv gesetzt.

2 Um etwaige semantische Verschiebungen zu vermeiden, werden die Subkategorien der Kategorie *Thematische Forschungsschwerpunkte* im Folgenden auch in der Originalsprache (Englisch) aufgeführt.
